# Epigenetic Alterations in Parathyroid Cancers

**DOI:** 10.3390/ijms18020310

**Published:** 2017-02-01

**Authors:** Chiara Verdelli, Sabrina Corbetta

**Affiliations:** 1 Laboratory of Experimental Endocrinology, IRCCS Istituto Ortopedico Galeazzi, 20161 Milan, Italy; chiara.verdelli@libero.it; 2 Endocrinology Unit, Department of Biomedical Sciences for Health, University of Milan, IRCCS Istituto Ortopedico Galeazzi, 20097 Milan, Italy

**Keywords:** parathyroid cancers, parathormone (PTH), DNA methylation, histones, methyltransferases, microRNAs

## Abstract

Parathyroid cancers (PCas) are rare malignancies representing approximately 0.005% of all cancers. PCas are a rare cause of primary hyperparathyroidism, which is the third most common endocrine disease, mainly related to parathyroid benign tumors. About 90% of PCas are hormonally active hypersecreting parathormone (PTH); consequently patients present with complications of severe hypercalcemia. Pre-operative diagnosis is often difficult due to clinical features shared with benign parathyroid lesions. Surgery provides the current best chance of cure, though persistent or recurrent disease occurs in about 50% of patients with PCas. Somatic inactivating mutations of *CDC73/HRPT2* gene, encoding parafibromin, are the most frequent genetic anomalies occurring in PCas. Recently, the aberrant DNA methylation signature and microRNA expression profile have been identified in PCas, providing evidence that parathyroid malignancies are distinct entities from parathyroid benign lesions, showing an epigenetic signature resembling some embryonic aspects. The present paper reviews data about epigenetic alterations in PCas, up to now limited to DNA methylation, chromatin regulators and microRNA profile.

## 1. Introduction

Tumors of the parathyroid glands frequently occur, showing a prevalence of 0.1%–0.4% in the general population, increasing up to 4% in postmenopausal women [1]. Parathyroid tumors are often associated with parathormone (PTH) hypersecretion determining primary hyperparathyroidism (PHPT), which represents the third most common endocrine disease following diabetes and thyreopathies. In PHPT, PTH hypersecretion due to tumor parathyroid cell proliferation induces hypercalcemia by increasing calcium mobilization from bone and calcium reabsorption from kidney. Most tumors of parathyroid glands are benign lesions, involving one or multiple parathyroid glands. Parathyroid benign tumor-related morbidity comes from PTH inappropriate and uncontrolled release.

Similarly to other human cancers, parathyroid carcinomas (PCas) display local vascular and tissue invasiveness, as well as metastatic localization. At variance with the most common human cancers, PCas are rare, accounting for approximately 0.005% of all cancers. Moreover, precancerous lesions, potentially evolving in cancer, have not been identified in parathyroid glands. These peculiarities suggest that parathyroid cells are highly resistant to cancer, though parathyroid cells are stimulated by several conditions, whose persistence induces cell proliferation: (1) pregnancy increases the rate of PTH release in the first trimester to face the embryonic needs; (2) chronic calcium and vitamin D deficiencies induce benign proliferative cell response; (3) congenital inactivating mutations of the calcium sensing receptor (*CASR*) gene are associated with parathyroid glands hyperplasia. However, none of these conditions are associated with PCa development.

Parathyroid cancers occur both as part of familial syndromes or sporadic cases. PCas can be diagnosed in 15% of patients affected with hyperparathyroidism-jaw tumor (HPT-JT) syndrome (Online Mendelian Inheritance in Man-OMIM#145001), harboring inactivating mutations of the *HRPT2/CDC73* gene (reviewed in [2]). HPT-JT patients develop PCas or solitary, rarer multiple, cystic parathyroid adenomas. PCas have been occasionally reported also in patients affected with multiple endocrine neoplasia type 1 syndrome (OMIM#131100), harboring inactivating mutations of the *MEN1* gene. MEN1 patients usually develop parathyroid benign hyperplasia [2]. Somatic inactivating mutations of the *HRPT2/CDC73* and *MEN1* genes occur also in sporadic PCa cases.

Most of the clinical and molecular studies investigated parathyroid tumor series, including both benign and malignant cases, often revealing a consistent overlap of the clinical, genetic and epigenetic features between the two entities. Nonetheless, benign and malignant parathyroid lesions differ in some genetic and epigenetic aspects. Herein, we focused the attention on PCas characterizing their clinical, genetic and epigenetic signatures.

## 2. Clinical Features of Parathyroid Cancers

Parathyroid carcinomas are usually hormonally functional, and patients with PCas are referred for clinical symptoms of hypercalcemia. Parathyroid hormone (PTH) release is often elevated, and severe hypercalcemia develops. Serum calcium often exceeds 14 mg/dL, and serum PTH is significantly elevated commonly between three- and 10-times the upper limit of the normal range. Most common hypercalcemia-related symptoms are quite unspecific: dehydration, hypotension, mental confusion, muscle weakness, nausea, anorexia, weight loss and constipation.

Preoperative diagnosis of parathyroid carcinoma is difficult unless patients present with metastatic disease. Fine needle aspiration is not recommended because of the risk of parathyromatosis [3]. The main challenge in histologic diagnosis consists of distinguishing atypical parathyroid adenomas from PCas. The two entities have different clinical behaviors reflected in the overall recurrence rates, disease-free survival and overall survival. Atypical parathyroid adenomas present with a less severe biochemical profile and demonstrate an indolent clinical course compared with PCas [4].

Treatment options include surgery, while postoperative radiotherapy remains controversial [3]. Indeed, PCas are usually radioresistant; thus, a protocol of radiotherapy has not been established [5]. Moreover, the standard chemotherapy regimen is not available, as chemotherapy is generally ineffective in the treatment of PCa [5]. Hypercalcemia-related symptoms can be controlled by cinacalcet HCl, a potent agonist of the calcium sensing receptor (CASR). Activation of CASR on parathyroid cells by cinacalcet inhibits PTH release [6]. Alternative medical approaches aimed to reduce hypercalcemia are bone antiresorptive agents, namely bisphosphonates and denosumab [7].

Survival analysis of PCa patients extrapolated from various cancer databases, such as Surveillance, Epidemiology, and End Results Program (SEER), National cancer Database (NCDB) and the Swedish Cancer Registry, and from longitudinal retrospective studies at single institutions showed an overall survival of 85% and 49%–77% after a follow-up of five and 10 years, respectively. Thus, the prognosis displays a better survival rate than most solid tumors. The recurrence rate is increased in those patients who manifest lymph node and/or distant metastases, while tumor size is not a prognostic factor; the mean-time of recurrence ranges between 2.5 and 4.8 years after the initial surgical intervention. Recurrences are mainly locoregional, and distant metastases occur in one fourth of patients. Common sites of metastases include lung, bone and liver.

Mortality is usually secondary to intractable hypercalcemia, which leads to progressive end-organ damage, such as renal failure. The natural progression of PCa is usually that of a slow-growing neoplasm with a long clinical course complicated by tumor recurrence, reoperations and progressive end-organ damage due to disturbed calcium homeostasis.

## 3. Genetic Features of Parathyroid Cancers

The genetics of PCas is characterized by loss of tumor suppressors, as suggested by frequent loss of DNA detected by comparative genomic hybridization (CGH) in about 40 PCas [8,9]. In these studies, the most frequently-detected imbalances were losses of chromosomes 1p, 13q and 17; in particular, commonly-occurring DNA losses were 1p21-p22 (41%), 13q14-q31 (41%), 9p21-pter (28%), 6q22-q24 (24%), 4q24 (21%) and 17 (30%), whereas gains preferentially involved 19p (45%), Xc-q13 (28%), 9q33-qter (24%), 1q31-q32 (21%), 5 (30%) and 16p (21%).

Whole-exome sequencing in seven PCas showed that PCas had an average of 51 somatic variants/tumor (range of 3–176) with approximately 58% of variants occurring as nonsynonymous single nucleotide variants [10]. Loss of the tumor suppressor *HRPT2/CDC73*, located at 1q31 and encoding for parafibromin, has been identified in HPT-JT-related PCas and in 70% of sporadic PCas [11], representing the most common genetic aberration so far identified in PCas. Mutant *CDC73* alleles are preferentially amplified [10]. Loss of parafibromin expression in PCa specimens occurs in about 50% of cases, and when associated with *CDC73* mutations, it predicts a lower overall survival of 10 years [12]. Moreover, losses of adjunctive tumor suppressors have been detected in PCas: inactivating mutations of *PRUNE2* [10] and *MEN1* [13] have been found in 18% and 13% of PCas, respectively. *PRUNE2* is located on chromosome 9q21.2 and encodes for a protein with several functions, including suppression of Ras homolog family member A (*RHOA*) activity, which results in reduced stress fiber formation and suppression of oncogenic cellular transformation. The tumor suppressor *MEN1* gene, located on chromosome 11q13 and encoding for menin, is lost in tumors of MEN1 syndrome and in about 30% of sporadic parathyroid adenomas. At variance with the most common human cancers, inactivating mutations of *RB1* or *P53* have not been identified in PCas [14,15,16].

## 4. DNA Methylation in Parathyroid Cancers

National and international mapping projects, such as those conducted by the U.S. National Institutes of Health (NIH) Roadmap Epigenomics Mapping Consortium [17], the International Human Epigenome Consortium [18], the Cancer Genome Atlas Network [19], European initiative to establish epigenomic maps of blood cells (BLUEPRINT) and the International Cancer Genome Consortium, have defined the genome-wide distribution of epigenetic markers in many fetal and adult normal and cancerous tissues. Unfortunately, no data about parathyroid normal or cancer tissues have been produced from the derived databases. Few studies have investigated epigenetic signature in PCas; however, available data provide interesting insights into parathyroid tumorigenesis. Here, we report these data attempting to define the landscape of epigenetic alterations in PCas.

### 4.1. Global DNA Methylation Pattern in Parathyroid Cancers

Gene silencing by DNA promoter cytosine phosphate guanine (CpG) islands methylation is the main and most well-studied epigenetic mechanism in humans, and it is intimately involved in cancer development [20]. DNA methylation is catalyzed by DNA methyltransferases (DNMTs), while the erasure of DNA methylation is achieved by ten-eleven translocation (TET) methylcytosine dioxygenases. TET enzymes convert 5-methylcytosine (5mC) to 5-hydroxymethylcytosine and subsequently to formyl or carboxyl cytosine. Then, modified cytosine is excised by thymine-DNA glycosylase (TDG) and repaired with an unmodified cytosine. The TET2 genes are frequently mutated in human cancers [21]. Spontaneous hydrolytic deamination of 5mC to thymine induces a high percentage of point mutations in germ or somatic cells, contributing to the genetic changes.

Comparing the DNA methylome profiles of seven metastatic PCas, whose *CDC73* status was not investigated, and three normal parathyroid glands, 175 differentially-methylated genes were identified, while comparing the methylome profiles of PCas with those of 14 parathyroid benign adenomas, 263 genes with distinct methylation levels were detected [22]. Considering the top 100 differentially-methylated CpG islands, PCas showed the hypermethylation of all examined CpG islands with a pattern clearly distinguishable from that detected in normal parathyroid glands, which displayed low levels of CpG methylation. However, global hypermethylation were not detected in three PCas harboring *CDC73* mutations analyzed using long interspersed nucleotide element-1 (LINE-1), a surrogate marker of genome-wide methylation changes [23]. Indeed, LINE-1 has been considered more sensitive in detecting decreases in DNA methylation rather than increases [24]. The authors admitted that the discrepancy could be explained by the different position of the analyzed CpG dinucleotides, the tumor heterogeneity, as well as the cut-offs and algorithms used for the detection of differential methylation used in both methods.

In line with the global hypermethylation detected by Starker and collaborators, a recent study reports a consistent reduction of the global 5-hydroxymethylcytosine (5hmC) levels in the analyzed PCas compared to parathyroid normal glands [25]. All 17 PCas stained negatively for 5hmC, as well as for TET1.

### 4.2. Regional DNA Methylation

Quantification of the methylation density by the pyrosequencing technique at the promoter of several candidate genes in *CDC73*-mutated PCas [23] showed that *APC*, *SFRP1* and *RASSF1A* were hypermethylated, while *CTNNB1*/β-catenin was not affected. These findings confirmed previously reported hypermethylation of the *APC* gene promoter associated with reduced *APC* mRNA and protein expression levels and with increased levels of active unphosphorylated β-catenin in five PCas [22,26]. Other genes regulating the Wnt/β-catenin pathway have been found hypermethylated in PCas: *SFRP1* [22,23], a potent antagonist of the Wnt signaling pathway, *SFRP4* and *SFRP2* [22]. It is worth noting that *SFRP1* silencing by methylation can constitutively activate the Wnt signaling pathway. Furthermore, rat sarcoma (RAS) association family domain 1 (*RASSF1A*) is an important tumor suppressor gene involved in the regulation of cell growth and proliferation, as well as DNA repair and hypermethylation were frequently reported in many human cancers [27]. Hypermethylated genes in PCas included also [22]: (1) tumor suppressor genes involved in the cell cycle control, such as *CDKN2B*/p15INK4b, *CDKN2A*/p16INK4; (2) genes involved in apoptosis, such as *PYCARD*, *SOCS3*; (3) phosphatases, such as *DUSP8* and *PTPN20*; (4) transcription factors, including *HOXC11*, *WT1*, *GATA4* and *HIC1* [28]. In particular, *HIC1* encodes a transcriptional repressor shown to participate in complex regulatory loops resulting in increased p53 activation and inhibition of E2 transcription factor 1 (E2F1) through direct and indirect interactions with sirtuin 1 (SIRT1). In PCas, hypermethylation of *HIC1* is associated with reduced gene expression, due to repressive histone H3K27me2/3 modifications induced by the polycomb repressor complex 2 (PRC2) member Enhancer of Zeste Homolog 2 (EZH2) [28].

No *MEN1* or *CDC73* promoter hypermethylation could be detected in PCas [23,29,30], despite a previous study reporting methylation of the *CDC73* gene promoter in two out of 11 PCa samples [31]. These findings suggest that the mutational status of these two genes is unlikely to direct the tumors toward a different methylation profile. Similarly, no alteration of the CpGs methylation has been detected in the promoter region of the calcium sensing receptor (*CASR*) and vitamin D receptor (*VDR*) genes, two key molecules of parathyroid cells, conferring the sensitivity to extracellular calcium and precociously downregulated in parathyroid tumors [32,33].

A minority of aberrant methylated genes in PCas compared with normal parathyroid glands displayed hypomethylation [22]. In particular, hypomethylation of the promoter region of the microRNAs cluster on chromosome 19 (C19MC) has been detected in more than a half of PCas, though it did not correlate with C19MC microRNA expression levels [34].

**Figure 1:**
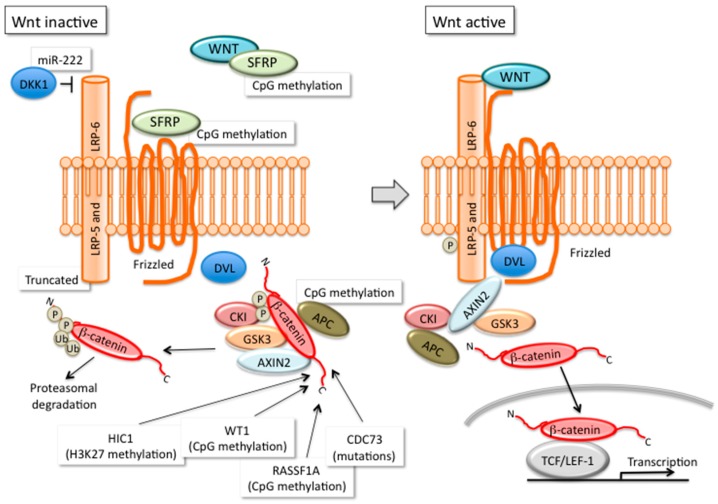
The Wnt/β-catenin pathway is potentially deregulated in PCas. Schematic representation of the molecules involved in the Wnt/β-catenin signaling in the inactive (left) and active (right) state: molecules, whose expression may be affected by genetic and epigenetic modifications in PCas, are indicated. The Wnt/β-catenin deregulation has been suggested as a “hub” of parathyroid tumorigenesis [37]. DKK1, Dickkopf 1; DVL, disheveled segment polarity protein; SFRP, secreted frizzled related protein; CKI, cyclin-dependent kinase inhibitor; GSK3, glycogen synthase kinase 3 β; TCF/LEF1, transcription factor 7/lymphoid enhancer binding factor 1; HIC1, hypermethylated in cancer 1; WT1, Wilms tumor 1; RASSF1A, Ras-association domain family 1; LRP-5, low density lipoprotein (LDL) receptor related protein 5; LRP-6, low density lipoprotein (LDL) receptor related protein 6; CDC73, cell division cycle 73; APC, WNT signaling pathway regulator; WNT, wingless-type ; ub, ubiquitination; ⊥, inhibitory effect.

The available data, summarized in Table 1, highlighted a specific “methylome” in PCas:

(1) PCas are characterized by hypermethylation rather than reduced levels of methylation, consistent with the loss of tumor suppressor genes as a hallmark of parathyroid tumorigenesis;
(2) All of the hypermethylated genes in PCas are hypermethylated also in benign parathyroid tumors; indeed, in PCas, the hypermethylation levels are more consistent;
(3) PCas show hypermethylation of the promoter region of genes commonly hypermethylated in human cancers, namely *CDKN2B*/p15, *CDKN2A*/p16, *SFRPs*, *RASSF1*, *HIC1* and *APC* [35];
(4) The promoter regions of the tumor suppressor genes known to be involved in parathyroid tumorigenesis, namely *CDC73*, *MEN1*, *CASR* and *VDR*, are not affected by increased methylation; therefore, methylation is not the major molecular mechanism inducing their loss in parathyroid tumor cells;
(5) Hypermethylation affects the promoter of genes encoding molecules of the Wnt/β-catenin pathway (Figure 1). Wnt/β-catenin is potentially deregulated in PCas by loss of parafibromin, which is a member of the polymerase II complex interacting with β-catenin [36]. Therefore, Wnt/β-catenin deregulation has been suggested as a “hub” of parathyroid tumorigenesis [37] (Figure 1). Indeed, accumulation of β-catenin is controversial in PCas, with studies reporting constitutive accumulation of active unphosphorylated β-catenin [26] and others failing in the detection of total β-catenin at the nuclear level [38].

### 4.3. Chromatin Regulators in Parathyroid Carcinomas

The genome sequencing efforts of thousands of uncultured tumors have revealed the frequent existence of mutations in writers, readers and erasers, thus establishing a causative role for an altered epigenome in cancer. Here, aberrant expression of chromatin regulatory molecules identified in PCas are reported.

#### 4.3.1. Histones Modifications in Parathyroid Cancers

Replication-dependent histones are tightly regulated throughout the cell cycle. Interestingly, histone expression is mainly regulated by the processing of the 3′ end of histone transcripts.

**Figure 2:**
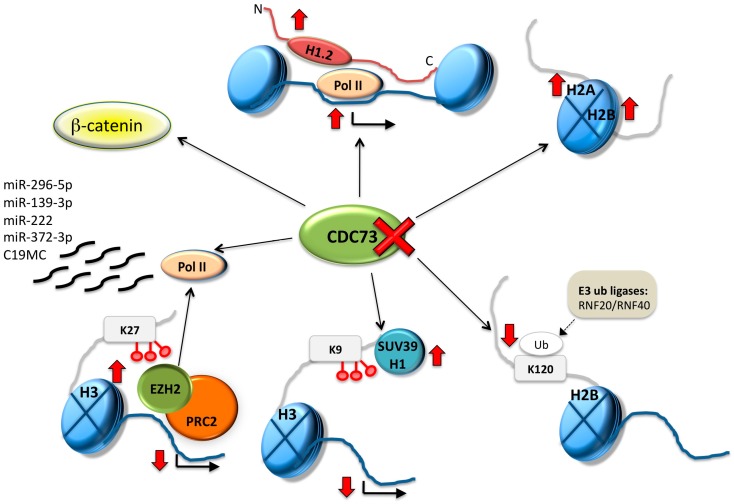
Effects of the loss of cell division cycle 73 (CDC73)/parafibromin on epigenetic regulatory mechanisms in PCas. Direct and indirect interactions between CDC73/parafibromin and histone H1.2, H2A, H2B, SUV39H1, EZH2 and microRNAs biogenesis are represented; moreover, CDC73/parafibromin may modulate the Wnt/β-catenin pathway in PCas. H, histone; Pol II, RNA polymerase II; Ub, ubiquitination; K, lysine; RNF, ring finger protein; SUV39H1, suppressor of variegation 3–9 homolog 1; PRC2, polycomb repressive complex 2; EZH2, enhancer of zeste 2; dashed arrow, supposed, not demonstrated, effect; red thick arrow, increased or decreased expression levels; red cross, loss of expression.

In the absence of parafibromin in *CDC73*-mutated PCas, replication-dependent histone transcripts are not cleaved and contain a poly(A) tail. Loss of parafibromin in HCT116 cells and HeLa cells upregulates the replication-dependent histone family; of these, histones *H1*, *H2A*, *H2B* and *H3* mRNAs are increased. The abnormally polyadenylated histone transcripts display an increased mRNA stability. Therefore, parafibromin emerges as a regulator of a posttranscriptional pathway critical to cell-cycle progression [39]. In line with these observations, histone H1.2 (histone cluster 1 H1 family member c; *HIST1H1C* (*6p22.2*)) has been reported up-regulated and highly overexpressed in seven sporadic PCas, together with the upregulation of histone H2A (*HIST1H2AC* (*6p22.2*)) and histone H2B (*HIST1H2BC* (*6p22.2*)*; HIST1H2BD; HIST1H2BK; HIST1H2BO*) [40] (Figure 2). These genes belong to the histone microcluster on chromosome 6p22.2-p21.3.

Moreover, in PCas there is loss of monoubiquitinated H2B at lysine 120 (K120)(H2Bub1). Parafibromin is required for the maintenance of H2B-K120 monoubiquitination [41] (Figure 2), while the level of H2B is consistently high in all parathyroid tumors independent of *CDC73* expression. H2Bub1 is involved in RNA elongation, while losses of H2Bub1, as well as of nuclear *CDC73* expression do not affect H3K4me3.

#### 4.3.2. Aberrant Expression of Methyltransferases in Parathyroid Cancers

Unmethylated CpG islands are key factors in controlling H3K4me3 levels through recruitment of H3K4 methyltransferases. CpG islands likewise play an important role in establishing and maintaining H3K27me3 at bivalent domains. H3K27me3, a pivotal marker in the establishment of repressive chromatin in both early development and adult organisms, is activated by the polycomb group (PcG) PCR2. The histone 3 lysine 27 methyltransferase Enhancer of Zeste Homolog 2 (EZH2) is a member of PCR2. *EZH2* mRNA and protein are overexpressed in PCas due to gene amplification [42], while *EZH2* mutations have not been detected in 23 sporadic PCas [43]. EZH2 is involved in H3K27 methylation, and EZH2-mediated epigenetic control of RNA polymerase II (Pol II) transcribed coding gene transcription has been well established [44,45] (Figure 2). Moreover, the histone methyltransferase SUV39H1 is potentially deregulated in PCas as its recruitment and induction of H3K9 methylation are dependent on parafibromin [46] (Figure 2). Unfortunately, SUV39H1 expression and function have not been investigated so far in PCas.

Considering these limited data, PCas may be supposed to have elevated global H3K27me3 levels. Indeed, increased EZH2 activity redistributes the H3K27me3 marker across the genome with a complex effect on transcription, including a loss of H3K27me3 that is associated with increased transcription at many loci [47].

## 5. MicroRNAs Deregulated in Parathyroid Cancers

MicroRNAs (miRNAs), single-stranded non coding RNAs of 19–25 nucleotides in length, are drivers or suppressors of the hallmarks of malignant cells [48]. In general, cancer cells show a large alteration of miRNA expression compared to their normal counterpart. All tumors show a specific miRNA signature, referred to as “miRNome”. Each miRNA is predicted to repress the expression of thousand mRNAs, but, in turn, each mRNA can be targeted by several hundreds of different miRNAs. Currently, there are about 1800 annotated human miRNA precursor genes that are processed into more than 2500 mature miRNA sequences (http://www.mirbase.org).

miRNA signature has been investigated in PCas compared with normal parathyroid glands [34,49,50] and with parathyroid benign tumors [34,50]. These studies identified a global miRNA downregulation in PCas compared to normal parathyroid glands, reflecting a deregulated pattern common to human cancers. Nonetheless, the different technical strategies employed produced different sets of significantly deregulated miRNAs. Among the downregulated miRNAs, miR-296-5p [49], miR-139-3p [49], miR-126-5p [50], miR-26b [50] and miR-30b [50] were the most significantly varied in PCas. Few miRNAs were upregulated in PCas; the most significantly varied were miR-222 [49,50], miR-503 [49] and miR-517c [34,49] (Table 2).

PCa deregulated miRNAs could be detected with a similar pattern of expression in their distant metastasis: miR-296-5p was downregulated in lung metastasis of a PCa [34], and C19MC miRNAs and miR-372 were upregulated in samples derived from five PCa metastases [34].

A number of molecular mechanisms may deregulate miRNAs expression in PCas. Loss of parafibromin, which interacts with RNA polymerase II (Pol II) [51], may potentially alter miRNAs expression, being a key molecule in miRNA transcription (Figure 2). Unfortunately, the relationship between loss of parafibromin in PCas and miRNAs expression have not been investigated so far. The transcriptional repression of miRNAs by hypermethylation of their corresponding promoter loci is a common feature of all human tumors [52]. Interestingly, PCas are characterized by the deregulation of microRNAs belonging to methylated genomic regions, namely the C19MC, miR-371-373 and GNAS loci.

*C19MC* cluster: PCas showed miR-519d, miR-518e and miR-517c median expression levels comparable with those in human placentas, the only human adult tissue physiologically expressing C19MC miRNAs. Two thirds of PCas showed gains in DNA copies either in the miRNA cluster regions or at a distant position along chromosome arm 19q, suggesting a genetic mechanism responsible for the aberrant expression of C19MC miRNAs at least in a subset of PCas [34]. Additionally, the C19MC promoter was hypomethylated in about half of PCa samples. However, the epigenetic status of the C19MC promoter was not correlated with miRNAs expression levels [34]. C19MC is the largest human miRNA gene cluster. It maps on chromosome 19q13.42, spanning about 100 kb and consisting of 46 genes encoding a total of 56 mature miRNAs [53]. miRNAs belonging to the C19MC cluster are expressed in human embryonic stem cells and rapidly downregulated during the differentiation process [54]. Expression of C19MC microRNAs increases significantly in trophoblasts from the first to third trimester of gestation [55], while they are silenced in the majority of adult normal cells by hypermethylation [56]. C19MC miRNAs are aberrantly expressed in infantile hemangioma [57], melanotic neuroectodermal tumor of infancy [58], embryonal tumors with multilayered rosettes [59], testicular germ cell tumors (in particular, in non-seminoma aggressive tumors) [60], glioblastoma [61], hepatic mesenchymal hamartomas [62], suggesting that C19MC reactivation characterizes tumors with embryonic features [63].

*miR-372*: miR-372, which is highly expressed in almost all PCas, belongs to the miR-371-373 cluster on chromosome 19q13.42, which is close to the C19MC cluster. Similarly to C19MC miRNAs, the miR-371-373 cluster is an “embryonic” cluster. miR-372 is deregulated in many human cancers [64].

*miR-296*: miR-296-5p is located at the imprinted locus *GNAS* (guanine nucleotide binding protein (G protein), α stimulating activity polypeptide 1) on chromosome 20q13. miR-296-5p is expressed from the paternally-derived allele, arising from the long, noncoding antisense transcript, GNAS antisense RNA 1 (*GNAS-AS1*) [65]. It is a tumor suppressor in breast and lung cancers [66,67]. In PCas, miR-296-5p is downregulated, showing the best predictive value in distinguishing cancers from normal parathyroid glands [49].

## 6. Concluding Remarks

Though epigenetic investigation in PCas is limited and needs further studies, available data provide intriguing perspectives and suggest new approaches in parathyroid tumorigenesis research; the main remarks are:

(1) Epigenetic studies increased the list of tumor suppressor genes lost in parathyroid cancers (Table 1), suggesting that loss of tumor suppressor genes, rather than gain of oncogenes is a hallmark of parathyroid tumorigenesis;
(2) Parafibromin, the most common tumor suppressor lost in PCas, emerges as a coordinator regulating a number of different pathways: (1) Wnt/β-catenin; (2) chromatin remodeling; (3) miRNA transcription (Figure 2);
(3) Most deregulated miRNAs in PCas are not exclusive of parathyroid cells, since they characterize the miRNA signature of most common human cancers. As an example, the miR-222/221 cluster is one of the most commonly upregulated miRNAs in human cancers. However, it is of interest to consider that they belong to methylated loci and are common in the human embryonic stem cell miRNA signature;
(4) Distant PCa metastases usually share the miRNA expression profile of the primitive tumors; no data are available about their methylome signature.

## Figures and Tables

**Table 1:** Aberrant gene expressions and related molecular mechanisms in parathyroid carcinomas compared with normal parathyroid glands.

Gene ID	Chr. Map	Gene Function	Variation in PCas	Frequency in PCas
**Loss of function mutated genes**				
*CDC73*	1q31.2	Tumor suppressor involved in transcriptional and post-transcriptional control	⇩	70%
*PRUNE2*	9q21.2	Tumor suppressor involved in the suppression of Ras homolog family member A activity	⇩	18%
*MEN1*	11q12	Tumor suppressor associated with MEN1 syndrome	⇩	13%
**Hypermethylated genes**				
*HIC1*	17p13	Tumor suppressor involved in inhibition of E2F1 through interaction with SIRT1	⇩	100%
*APC*	5q22.2	Tumor suppressor, inhibitor of the Wnt/β-catenin pathway	⇩	33%–100%
*RASSF1A*	3p21.31	Ras-association domain family 1	⇩	100%
*SFRP1*	8p11.21	Secreted frizzled related protein 1, inhibitor of the Wnt/β-catenin pathway	⇩	100%
*SFRP2*	4q31.3	Secreted frizzled related protein 2, inhibitor of the Wnt/β-catenin pathway	⇩	n.a.
*SFRP4*	7p14.1	Secreted frizzled related protein 4, inhibitor of the Wnt/β-catenin pathway	⇩	n.a.
*CDKN2A/p16*	9p21.3	Cyclin-dependent kinase inhibitor	⇩	n.a.
*CDKN2B/p15*	9p21.3	Cyclin-dependent kinase inhibitor	⇩	n.a.
*WT1*	11p13	Wilms tumor 1, tumor suppressor	⇩	n.a.
**Hypomethylated genes**				
*C19MC*	19q13.42	microRNA cluster	⇧	58%
**Aberrantly expressed chromatin remodeling genes**				
*HIST1H1C*	6p22.2	Replication-dependent histone H1.2	⇧	100%
*HIST1H2AB*	6p22.2	Replication-dependent histone H2A	⇧	n.a.
*HIST1H2BB*	6p22.2	Replication-dependent histone H2B	⇧	n.a.
*EZH2*	7q36.1	Enhancer of zeste 2 polycomb repressive complex 2 subunit	⇧	100%

Chr., chromosome; PCas, parathyroid carcinomas; CDC73, cell division cycle 73; APC, Wnt signaling pathway regulator; PRUNE2, prune homolog 2; MEN1, multiple endocrine neoplasia type 1 syndrome; HIC1, hypermethylated in human cancers 1; SIRT1, sirtuin 1; C19MC, chromosome 19 microRNA cluster; n.a., not available.

**Table 2:** Aberrant microRNA expressions in parathyroid carcinomas compared with normal parathyroid glands.

Gene ID	Chr. Map	Gene Function	Variation in PCas	Frequency in PCas
*miR-517c*	19q13.42	C19MC microRNA cluster	⇧	100%
*miR-372-3p*	19q13.42	miR-371-373 cluster	⇧	75%
*miR-139-5p*	11q		⇩	n.a.
*miR-296-5p*	20q13.32	*GNAS* imprinted locus	⇩	n.a.
*miR-503*	Xq26.3		⇧	n.a.
*miR-222*	Xp11.3	miR-221/222 cluster	⇧	n.a.
*miR-126-5p*	9q34.3		⇩	n.a.
*miR-26b*	2q35		⇩	n.a.
*miR-30b*	8q24.22		⇩	n.a.

C19MC, chromosome 19 microRNA cluster; GNAS, guanine nucleotide binding protein α stimulating complex locus; n.a., not available.
